# Immobilized nanoparticles-mediated enzyme therapy; promising way into clinical development

**DOI:** 10.1186/s11671-023-03823-7

**Published:** 2023-03-23

**Authors:** Ali Rajabi Zangi, Ala Amiri, Fatemeh Borzouee, Rafieh Bagherifar, Pouya Pazooki, Hamed Hamishehkar, Yousef Javadzadeh

**Affiliations:** 1 Student Research Committee, Tabriz University of Medical Sciences, Tabriz, Iran; 2 Department of Pharmaceutics, Faculty of Pharmacy, Tabriz University of Medical Sciences, Tabriz, Iran; 3 Department of Biotechnology, Faculty of Biological Sciences, Alzahra University, Tehran, Iran; 4 Department of Clinical Biochemistry, School of Pharmacy and Pharmaceutical Sciences, Isfahan University of Medical Sciences, Isfahan, Iran; 5 Cellular and Molecular Biology Research Center, Shahid Beheshti University of Medical Sciences, Tehran, Iran; 6 Drug Applied Research Center, Tabriz University of Medical Science, Tabriz, 5166-15731 Iran; 7 Biotechnology Research Center, and Faculty of Pharmacy, Tabriz University of Medical Science, Tabriz, 5166-15731 Iran

**Keywords:** Enzyme, Immobilization, Nanoparticles, Delivery system, Clinical development

## Abstract

Enzyme (Enz)-mediated therapy indicated a remarkable effect in the treatment of many human cancers and diseases with an insight into clinical phases. Because of insufficient immobilization (Imb) approach and ineffective carrier, Enz therapeutic exhibits low biological efficacy and bio-physicochemical stability. Although efforts have been made to remove the limitations mentioned in clinical trials, efficient Imb-destabilization and modification of nanoparticles (NPs) remain challenging. NP internalization through insufficient membrane permeability, precise endosomal escape, and endonuclease protection following release are the primary development approaches. In recent years, innovative manipulation of the material for Enz immobilization (EI) fabrication and NP preparation has enabled nanomaterial platforms to improve Enz therapeutic outcomes and provide low-diverse clinical applications. In this review article, we examine recent advances in EI approaches and emerging views and explore the impact of Enz-mediated NPs on clinical therapeutic outcomes with at least diverse effects.

**Graphical abstract:**

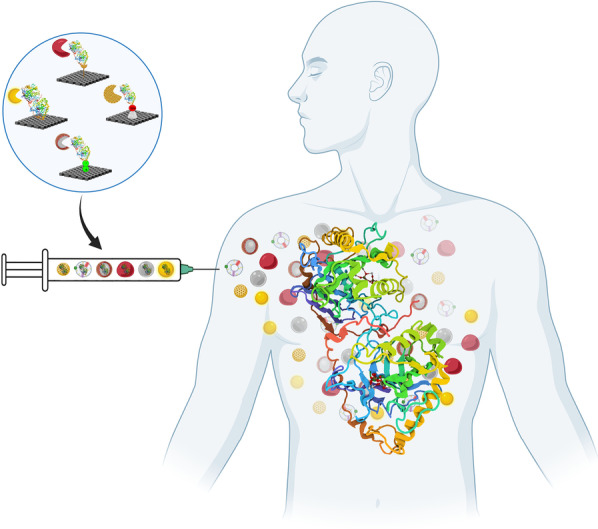

## Introduction

In recent years, therapeutic usage of an enzyme (Enz) has become a rapidly growing field due to the emergence of new clinical Enzs and the possible isolation in the pure form. Enz-mediated therapies are attracting increasing attention for the treatment of diseases caused by Enz misfolding, mutation, and deficiency, like amyotrophic lateral sclerosis [1]. The development of Enz therapeutics has led to restoring the biological function of Enzs related to abnormal clinical complications and delivery of normal Enzs in vivo or in vitro [2, 3]. Thus, it facilities an opportunity to manipulate cell functions, such as those affecting signaling pathways [4]. These Enzs have recently been used in cancer (gene) therapy, wound healing, and microbial infections [1].

Although Enz-based therapies offer the above benefits, they remain challenging due to hyperimmune responses and the intrinsic permeability and stability of the protein [5]. Additionally, intracellular delivery limits the effectiveness of treatments due to endosomal digestion and trapping [6, 7]. Already, two approaches have been developed to overcome these challenges, including the conjugation of Enzs to the cell-penetrating peptide (CPP) and Enz delivery using nanoparticles (NPs) as carriers. The first method has recently been proposed for Enz delivery. Nevertheless, it needs further development, while the second method is well-established. There are different platforms for the delivery of different Enzs.

Due to their low cytotoxicity and effectiveness in cell internalization, CPP, short cationic and amphipathic peptides (10–30 AA), can deliver molecular bioactive cargoes, such as Enzs. Attachment of Enzs to CPPs improves cellular uptake and endosomal escape, facilitates penetration of Enzs through the blood–brain barrier (BBB), and provides more effective approaches in the diagnosis and treatment of human diseases, such as diabetes, central nervous system disorders, inflammation, and cancer. However, given the potential of CPP conjugation, it seems that this area of research will be of more interest shortly.

NPs have revealed advantages over traditional bulk materials and received much attention in recent years as carriers of therapeutic molecules. These characteristics include small size, high surface-to-volume ratio, ease of surface modification, unique geometry, and size/shape-dependent characteristics. NPs can improve drug therapeutic and pharmacological features [8, 9]. The construction of NP-conjugates with therapeutic molecules (protein/Enz) is intended to protect them against degradation, prolong their presence in the human body, and reduce the cytotoxic property of the protein/Enz. The protection provides targeted delivery and reduces off-target side effects due to cargo-delivery capacity, cell-tracking signal, and targeting specific cells [10, 11]. Therefore, Enz-NPs delivery can provide an opportunity to treat these diseases derived from the lack of enzymatic activities [12]. To this end, the virus-like NPs delivered Cytochrome P450 (CYP) activity into tumor cells [13, 14]. The results demonstrated that increased CYP activity in the tumor tissues improved the treatment efficiency and reduced the dose and side effects. This NPs as tool for drug delivery systems (DDS) is not only restricted for cancers [5] but also cover many various diseases, including galactosemia, phenylketonuria, methylmalonic acidemia, homocystinuria, the deficit of carbamoyl phosphate synthetase I, argininosuccinic aciduria, argininemia, alkaptonuria, albinism and finally the congenital adrenal hyperplasia [12]. This review explores the immobilization (Imb) of Enz and provides insight into the current progress of nanodelivery platforms for therapeutic Enzs.

## Enzyme immobilization (EI)

Overall, immobilized Enzs on NPs indicate certain advantages over their free counterparts, such as improved targeting of specific tissues and cells, low immune response, high retention in the bloodstream, and high permeability. Therefore they represent more thermal stability, regulated pH tolerance, excellent storage stability, lower Km (the affinity constant of the Enz for its substrate), good reusability, easy separation from the reaction mixture, and minimum reaction product contamination to Enz in the industry, especially in EI [15–17]. Caicai Fu et al. co-immobilized glucose oxidase (GOx) and Fe_3_O_4_ NPs on a flower-shaped covalent organic framework (COF) to efficiently remove mycotoxins. The GOx-Fe_3_O_4_@COF hybrid catalyst exhibits excellent activity in mycotoxin removal due to the enrichment of mycotoxins in COF and the cooperative catalysis between GOx and Fe_3_O_4_ NPs. The degradation efficiency of aflatoxin B1 (AFB1) in the chemoenzymatic cascade reaction catalyzed by Fe_3_O_4_@COF (pH 3.0–7.0) is 3.5 times higher over in the Fenton reaction catalyzed ones (below pH 3.0) [18].

In another study, the 11-mercaptoundecanoic acid (11MUA) functionalized magnetic core–shell NPs (Fe_3_O_4_@Au-MUA NPs) were used to improve the properties of L-asparaginase (L-ASP) and to increase the anticancer effects of the cold atmospheric pressure plasma (CAPP). Immobilized L-ASNase showed slight changes in the kinetics parameters of V_max_ (from 227.27 ± 24.5 to 175.43.25.6 μM min^−1^ mg^−1^) and a decrease in K_m_ (from 5.45 ± 0.18 to 4.052 ± 11 mM). MTT assay showed that CAPP combined with L-ASNase immobilized onto the Fe_3_O_4_@Au had the highest anticancer effect against cancer cells, which decreased the viability of these cells by 43% [17].

Finally, a similar result with matrix metalloprotease 2 (MMP-2) responsive size-switchable UAMSN@Gel-PEG NPs (ultrasmall amino-modified mesoporous silica NPs wrapped within PEG-conjugated gelatin) was developed to deliver and diffuse H_2_ to the deep part of tumors extracellular matrices (ECM) for effective gas therapy. The result showed simultaneous deep tumor penetration of NPs and H_2_ results in an evident suppression of tumor growth in a 4T1 tumor-bearing model without any apparent toxicity on normal tissues [19].

**Fig. 1:**
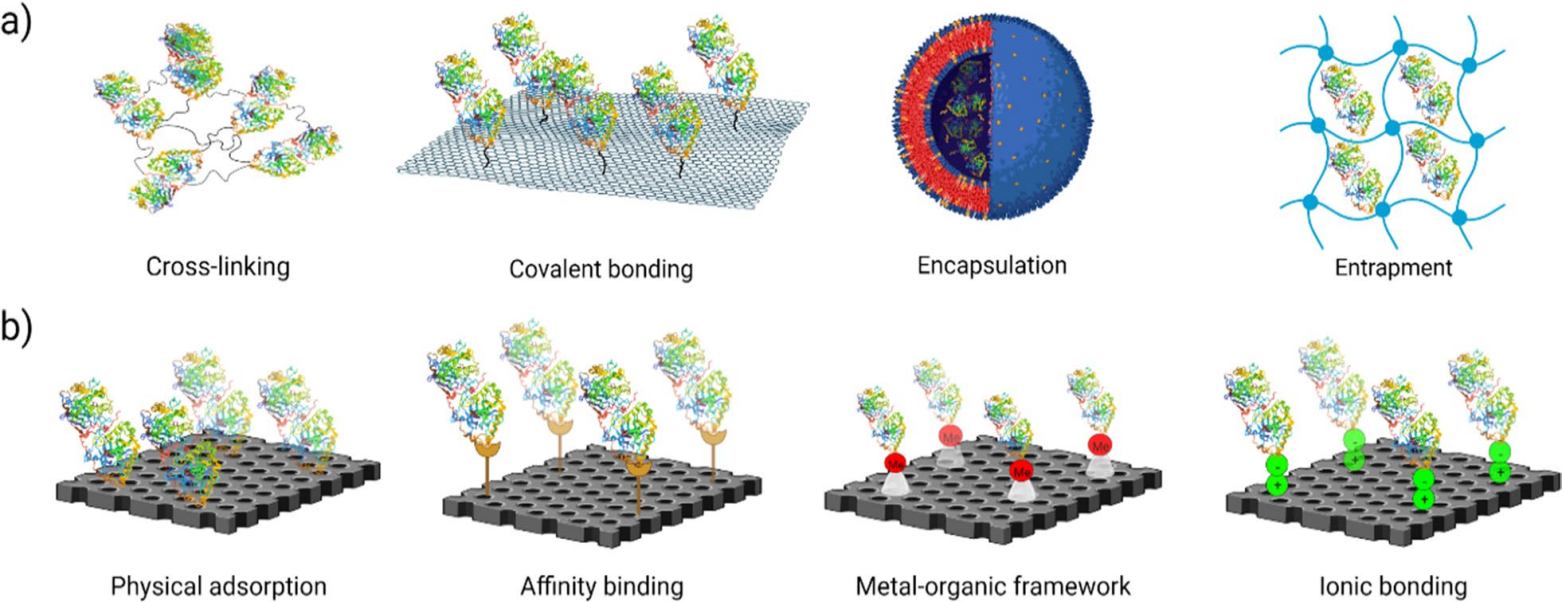
A representative view of the Enz immobilization with **a** chemical and **b** physical establishment approaches

EI on nanomaterials increases favorable interaction yielding and enhances Enz catalysis via several mechanisms. The mechanism is based on metal ion activation, electron transfer, temperature, and morphology effects of nanoscale supports, the multi-Enz system, and conformational changes of immobilized Enzs [20]. There are two main types of EI approaches: physical or reversible (physical adsorption, ionic bonding, affinity binding, and metal bonding) and chemical or irreversible (covalent bonding, entrapment, and cross-linking [21]. Physical methods are characterized by weaker, non-covalent interactions such as hydrogen bonds, hydrophobic interactions, van der Waals forces, affinity binding, and ionic bonding of the Enz with the material or mechanical containment of Enz within the solid support. In the chemical method, the formation of covalent bonds achieved through the ether, thioether, amide, or carbamate bonds between the Enz and supported material is involved [22]. Figure 1 displays a representation of the various EI methods with establishment type between cargo and supporting bed.

The selection of the EI method and the supporting material can affect the Imb process. It should not reduce Enz activity, whereby properties of both the Enz and the fixed material will dictate the EI approach. Therefore, understanding the role of amino acids at the Enz active site and knowing the matrix structure will help choose the proper stabilization method [22]. The composition, size, charge, surface characteristics, and chemical structure of the materials play critical roles in determining the efficiency of the immobilized system. Preferably, the support materials must prevent the Enz aggregation and denaturation but maintain the native structure of the Enz, and it should not interfere with the active site of the Enz [23, 24]. Also, understanding the role of amino acids at the Enz active site and knowing the structure of the matrix will help in choosing the proper stabilization method [25]. The method description and the pros and cons of EI methods are summarized in Table 1.

**Table 1:** The pros and cons of Enz immobilization methods

Immobilization methods		Method description	Advantages	Disadvantages	References
Physical method	Physical adsorption	Enzs are physically adsorbed or attached to the surface of support material through hydrogen bonds and weak non-specific forces (hydrophobic interactions, electrostatic, and van der Waals forces). Surface-bound arrangements are formed without any alteration in functionality	The reversible, broad range of carrier, minimum activation, economical and cost-effective, regeneration of support, high loading capacity, high Enz activity	Enz leakage by a change in temperature and ionic strength	[24]
	Ionic bonding	The principle of protein–ligand interaction rules includes an ionic in such a way that Enz to be bound, possesses an opposite charge	Simple, reversible, and the chances of Enz leakage are low	Low Enz activity	[26, 27]
	Affinity binding	Enz links to the matrix through specific interactions through covalent bonding (Enz is conjugated to an entity that develops an affinity towards the matrix), and the matrix is pre-coupled to an affinity ligand for target Enz	High selectivity, control orientation of the immobilized Enz, high binding capacity, and high reusability	Expensive	[28]
	Metal–organic framework	The metal ions (Ti and Zr), as viable host matrixes, are precipitated on the carrier (cellulose, chitin, alginic acid, and silica) surface. Metal chelated ions interact with N_2_ and O_2_ or with the nucleophilic groups (the imidazole of histidine, the indole of tryptophan, and the thiol of cysteine from Enz) on the matrix surface	Regeneration of Enz and support, high activity of the immobilized Enz	Enz leakage	[29]
Chemical method	Covalent bonding	Covalent bonding of Enzs to support matrix is typically occurring between the functional groups of support matrix and side-chain polar residues (arginine, lysine, aspartic and glutamic acids, histidine, cysteine, serine, and threonine), which are not essential for the catalytic activity of the Enz	Minimizes Enz leaching after repeated use	The inactivation of the Enz, requires complex activation steps	[29]
	Entrapment	Although in EI method Enzs are restricted by covalent or non-covalent bonds within a polymeric network, but substrates and the products are allowed to pass through it. Here, standard support like synthetic or natural organic polymers (polysaccharides, proteins, carbon vinyl, allyl polymers, and polyamides) and inorganic materials (activated carbon and porous ceramic) were utilized	Protects the Enz against proteases and microbial contamination, minimizes Enz leaching and improves the mechanical stability of the Enz	Limited Mass transfer, deactivation during the process, low loading capacity, and Enz leakage when the pore size is large	[30]
	Cross-linking	cross-linked Enz crystals (CLECs), cross-linked dissolved Enzs (CLEs), cross-linked Enz aggregates (CLEAs), and cross-linked spray-dried Enz (CLSD), do not use any support matrix and define as a covalent attachment of Enz molecules to each other by bi- or multi-functional reagents. Hence, insoluble aggregates with high molecular weight are formed, which are typically gelatinous	Minimizing leakage of Enzs, very concentrated Enz activity in the catalyst, the low production cost due to the removal of the carrier	It is very difficult to control, poor reproducibility, low mechanical stability, loss of Enz activity, the process is difficult	[31, 32]

## Nanoparticles-mediated Enz delivery

Therapeutic Enzs are one of the most promising applications in modern medicine due to their high affinity and specificity. However, it is costly to use them in clinical medicine because of their low bioavailability and low stability or lack of functionality in extreme pH, temperature, etc. [11, 33]. Therapeutic Enzs and proteins have received increasing attention since they carry out essential functions in various biological processes. The Enz delivery to intended sites is challenging due to Enzs intrinsic sensitivity to various conditions [1]. Intracellular delivery of non-functionalized Enzs is problematic since these proteins are not permeable to the cellular membrane. The transporting of cascade Enzs is even more problematic [34]. As a fraction of inclusion bodies, the transferred Enzs may not be active in the cells due to misfolding [35]. Hence, they need an appropriate delivery vehicle. The composition, size, charge, surface characteristics, and chemical structure of the materials play critical roles in determining the efficiency of the immobilized system. Preferably, the support materials must prevent the Enz aggregation and denaturation but maintain the native structure of the Enz, and it should not interfere with the active site of the Enz [36, 37].

**Fig. 2:**
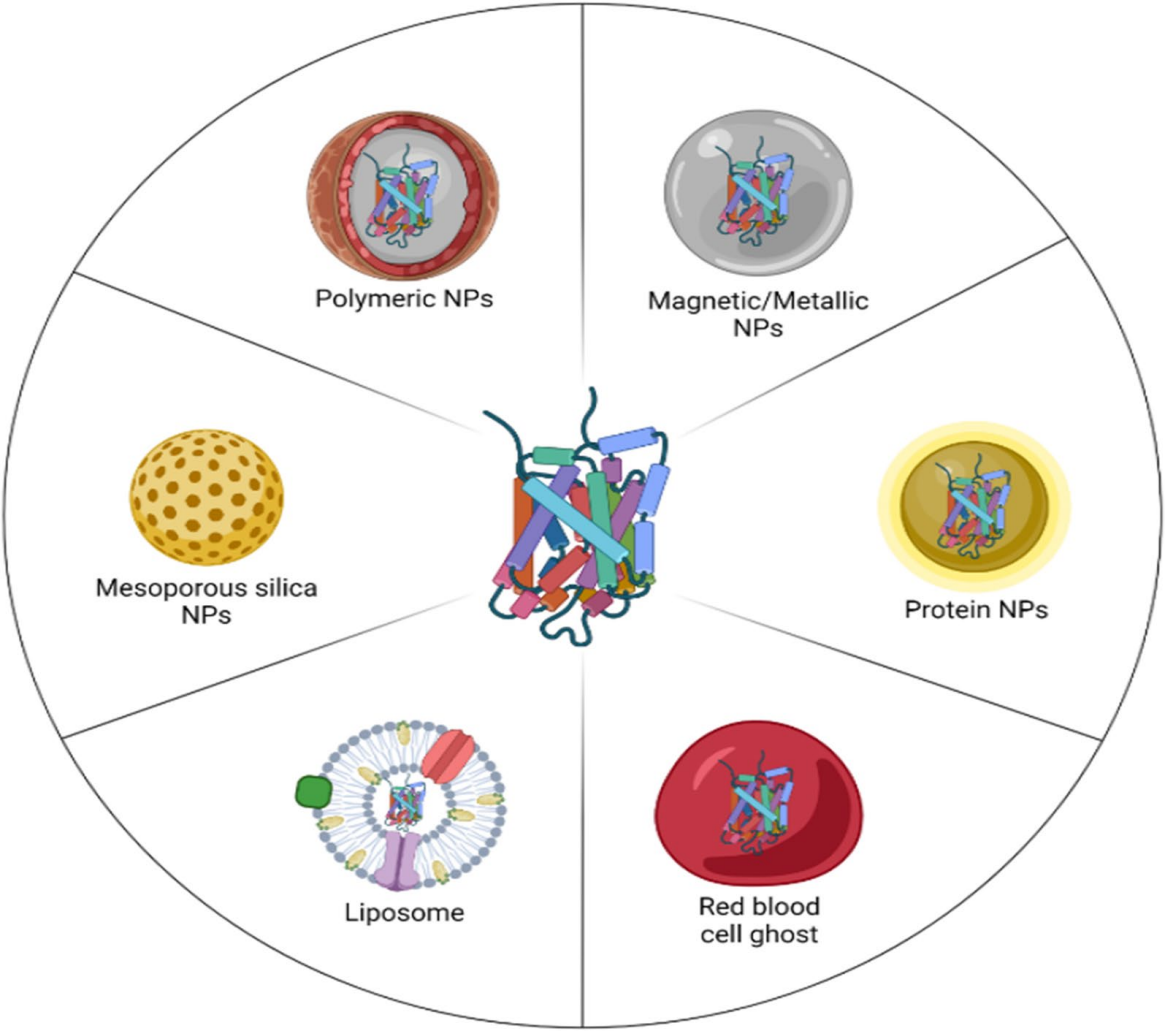
Schematic view of used nanoparticles for therapeutic Enz delivery

Liposomal, inorganic, polymeric, and biological NPs demonstrated remarkable benefits as Enz carriers [38]. A schematic view of used NPs for therapeutic Enz delivery illustrated in Fig. 2.

NPs can offer targeted delivery of proteins to particular locations and physical safekeeping from environmental stimuli [39]. Likewise, NPs can protect Enzs from denaturation or premature degradation in biological environments and increase the Enz half-life with poor pharmacokinetic (PK) properties in the systemic circulation [40]. As well as this, drug concentrations are maintained in the therapeutic window by regulating the sustained release. Finally, targeting disrupted intracellular compartments, damaged cells, and tissues improved the efficacy and safety of Enz-based therapeutics [5, 38]. Likewise, Steiert et al. indicate significant encapsulated lysozyme with a constant release through a non-toxic biopolymeric system to cells without the need for a chemical surfactant or cross-linker [41]. Furthermore, a recent study on the nanodelivery of functional Enz, demonstrated that the DNAzyme NPs down-regulate the early growth response factor-1 protein (EGR-1). This approach could prevent tumor cell growth and for inducing tumor cell apoptosis by aggregation-induced emission photosensitizer (TBD-Br) under light illumination [42]. The promising success of nanoformulation of Enzs such as glutathione peroxidase (GPX), asparaginase (ASP), serratiopeptidase (STP), β-galactosidase (βGL), and lysozyme (LYZ) has facilitated the development and manipulation of NPs for Enz delivery. The utilized NPs to mediate Enz regarding the type of NPs, Enz payloads, and the delivery feature of the delivery were summarized in Table 2.

**Table 2:** The type of nanoparticles, target, and result for enzyme delivery

Nanoparticle category	Nanoparticle types	Loaded enzyme	Target	Results	References
Inorganic NPs	MSN	SOD	Live cells	Enhancement of transmembrane delivery of SOD embedded in NPs	[43]
		GPX	HeLa cells	Provide antioxidant cascade reaction can be controlled through multifunctional MSN carrying antioxidant Enzs	[44]
		G6PD	–	Performance of EI with silica-based gels from different precursors efficiently	[45]
		P26S	HeLa cells	Internalize MSN into the host cell through clathrin- and caveolae-endocytosis and endosomal escape by the imidazole group	[46]
	Au NPs	βGL	COS-1, MCF7, and C2C12 cells	Efficient transfection (> 80%) of Gold NPs at 50 nM of Enz	[47]
	Nanocomplexes	AOX & CAT	Mice model	Alcohol prophylaxis and antidote for intoxications; thus, Enzs reduced blood alcohol levels in intoxicated mice	[48]
	Ag-silica hybrid NPs	α-Am	Kinetic tracking	Ag NPs play a key role in stabilizing the EI	[49]
	Ag NPs	Ly	HeLa cells	The nanobioconjugate showed synergistic antibacterial properties without damaging the catalytic site of Ly	[50]
		TRP	–	Ag-PDA conjugated trypsin was found suitable for efficient hydrolysis of different proteins in an industrial environment	[51]
		βKT	*Bacillus subtilis*	The application of βkT immobilized Ag NPs showed antibacterial potency and dehairing activity	[52]
	Cysteine-functionalized Ag	LIPA	–	The lipase NP bioconjugates showed a shelf life of two months with a marginal decrease in activity	[53]
	AgNPs doped gum acacia-gelatin-silica nanohybrid	Diastase (α-Am)		The kinetic parameters for the immobilized Enz included K_m_ = 10.3 mg/mL, V_max_ = 4.3 μmol/mL/min; however, for free Enz included K_m_ = 8.8 mg/mL, V_max_ = 2.8 μmol mL/min, indicating that the Imb improved the overall catalytic and stability property of the Enz	[54]
	MRNPs	CAT & SOD	Endothelial cells	The Enzs could be preserved from proteolytic degradation through encapsulation in MRNPs	[55]
	Fe_3_O_4_ MNPs	L-Asp	Jurkat E6-1 cells	The enzymolysis efficiency slightly increased with the polymer chain, resulting from the enhanced Imb amount of ASP	[56]
		βGL	–	The immobilized Enz showed higher activity and stability than its soluble counterpart as well as the magnetic-controlled positioning and reusability provided by coupling made this bioconjugate a good platform for the representation of highly pure and active GLA for both therapeutic applications and in vitro catalysis	[57]
		STP	Carrageenan-induced paw edema in rat	Targeting of MRNPs with immobilized Enz enhanced the delivery of the drug throughout the membrane in vitro and the increased anti-inflammatory impact in vivo at a much lower dose of Enz than the dose needed for treatment by using free STP	[58]
			–	Thrombolytic activity exhibited that the high thrombolytic activity can reach 92.8% for Nattokinase-NP conjugates and 208.7% for Lumbrukinase-NP conjugates much higher than the pure Enzs (NK, 83.8%; LK, 105.5%)	[59]
Polymeric NPs	PLGA	ASNase	Nasal and intestinal mucosae	Improved stability in the existence of LYZs and enhanced nasal transport of the protein	[60]
		SOD	Reperfusion injury mouse model	The NPs decreased the inflammatory markers and improved behavior in vivo	[61]
			Cultured human neurons	PLGA NPs were compatible with the neuron, and the neuroprotective impact of superoxide dismutase-poly PLGA NPs was dose-dependent, with high efficiency at > 110 U SOD	[62]
	Porous PLGA	TPA	–	Porous PLGA semi-IPN hydrogel was found useful as a local DDS to release active TPA primarily at the site of a thrombus	[63]
	Biodegradable PCL nanosphere	SOD and CAT	HaCat skin cells	An increase in SOD and CAT activities following enhanced uptake of PCL into the skin treatment, signifying the role of PCL-encapsulating CAT and SOD nanosphere in alleviating oxidative stress	[64]
	Nanocapsules	Tyr	Murine B16F10	Polyhb-Tyr inhibited the proliferation of murine B16F10 melanoma cells in the model of proliferation. It also inhibited the attachment of the cells in the model of attachment	[65]
Lipid based-NPs	Liposome	βGL	–	Entrapping in liposomes exhibited that all glucocerebroside beta-glucosidase activity can be incorporated in neutral egg phosphatidylcholine-cholesterol liposomes	[66]
		Papain	Rabbit model	In vitro and in vivo studies revealed PEL was proven as an excellent topical preparation for hypertrophic scar treatment	[67]
		RH-Cu/Zn- SOD	Thermally injured tissue of rabbit back skin	Local treatment of burn wounds with rh-Cu/Zn-SOD has a beneficial efficacy on the post-burn damage	[68]
		SOD	Sprague–Dawley rats	SOD encapsulated in liposomes had a beneficial effect on preventing the spread of post-burn injury	[69]
			Ear edema model	SOD-liposomes enable an edema inhibition of 65 ± 8%, over the 20 ± 1% of SOD in its free form	[70]
			Rheumatoid arthritis	Enzymosomes of SOD hydrophobic derivative had a more rapid anti-inflammatory impact than SOD liposomes, indicating the release of this SOD from liposome is no longer needed for achieving dismutation	[71]
	Chitosan-coated liposomes		Physicochemical evaluation	Highly efficient SOD-loaded vesicles for drug targeting on mucosal tissues	[72]
Biological NPs	Protein NPs	βGL	SK-BR-3 cells	> 80% of proteins were incorporated in the particles, and βGL activity was retained high after particle fabrication (> 75%)	[2]
		LYZ	Gram-positive *M. Luteus*	NPs retained the activity and structure of the LYZ, which was evaluated successfully against M. Luteus	[41]
		Bilirubin oxidase	Chronically jaundiced Gunn rats	In vivo administration of 0.1 to 2 mg/day of immobilized Enz over a four-day to rats effectively lowered plasma bilirubin level, but only when the molar ratio of B/RSA was more significant than 0.35	[73]
	Erythrocytes	AOX and ADH	Mice	Enz-loaded erythrocytes revealed an enhanced rate of hexose-monophosphate-shunt activity and methemoglobin production. In vivo, mice receiving AOX-loaded erythrocytes could keep the blood methanol below the value of around 50% of those uncovered in mice receiving unloaded cells and a similar amount of methanol	[74]
		PDE		Enz entrapment appeared as a method to inhibit and antagonize organophosphate poisoning	[75]
		TST	Human	Converted cyanides into less toxic thiocyanates	[76]
		Urikase		At a physiological concentration of uric acid, loaded erythrocytes could degrade around 23 mmol of uric acid/liter erythrocytes per min	[77]

*SOD* Superoxide dismutase, *GPX* Glutathione peroxidase, *G6PD* Glucose-6-phosphate dehydrogenase, *P26S* Proteasome 26S, *CAT* Catalase, *ASP* Asparaginase, *Tyr* Tyrosinase, *ASP* Asparginase, *TPA* Tissue plasminogen activator, *L-Asp* L-asparaginase, *SP* Serratiopeptidase, *SEP* Serine endopeptidase, Streptokinase, *AOX* Alcohol oxidase, *α-Am α*-amylase, *LYZ* Lysozyme, *TRP* Trypsin, *KRT* Keratinase, *LIPA* Lipase, *βGl* β-Glucosidase, *ADH* Alcohol dehydrogenase, *PDE* Phosphothioesterase, *TST* Thiosulfate-cyanide sulfurtransferase, *ALL* Acute lymphoblastic leukemia, *MSN* Mesoporous silica NPs, *βkT β*-keratinase, *PEL* Papain elastic liposomes, *RH* Recombinant Human, *MRNPs* Magnetically responsive NPs, *MNPs* Magnetic NPs, *PLGA* Poly(D, L-lactide-co-glycolide acid, *PCL* Polycaprolactone

## Inorganic nanoparticles

### Mesoporous silica NPs

As previously mentioned, NPs can serve as Enz carriers with a higher uptake/targeting mechanism, thereby overcoming the challenge of intracellular Enz delivery [78]. Enz delivery still needs efficient NPs to improve intracellular availability despite these advances. Among NPs, MSN is of interest because of their tunable surface chemistry, easily controllable morphology, well-defined pore structure, rigid framework, high drug loading capacity, and excellent biocompatibility [78]. Current progress in MSN, along with the presence of large pores, expands their application for protein delivery [79]. Besides, due to the possibility of abundant surface modifications, a variety of MSN-based stimuli-responsive systems with several benefits, such as release in specific targets, decreased toxicity, and increased efficacy, have been established [44, 80].

Direct delivery of antioxidant enzymes such as Zn/Cu-SOD into cells is a challenging task due to poor uptake. To overcome this cell barrier, MSN was established as a multifunctional vehicle to deliver SOD. The authors evaluated the functional recovery after ROS damage and the apoptosis level after free radical induction. It was found that the MSN-based delivery system has a non-endosomal distribution, and the transmembrane delivery of SOD was improved. Furthermore, the conjugation of CPP to MSN has been demonstrated to be a powerful strategy for improving the delivery of MSN to cells in molecular and intracellular therapy [43].

In another study, Han et al. produced proteasome-encapsulated MSN with Ni moieties (PtMSN), which proved to dissociate Tau insoluble aggregates, a pathological sign of Alzheimer complication. As a result, PtMSN could degrade the overexpressed Tau protein compared with the free proteasome and decrease the truncated isoform level, which is documented to be a cytotoxic aggregate [46]. One of the limitations of using traditional MSN in protein delivery is the small size of their pores, which may cause problems in Enz loading. Recently, surface-modified MSNs with large-sized pores and novel pore structures significantly deliver therapeutic agents [81].

#### Magnetically responsive NPs

Magnetically directed delivery can improve the therapeutic profiles of a wide range of pharmaceuticals by enhancing their release to the site of action while keeping off-target interaction [55, 58]. Consequently, MRNP encapsulates Enzs to facilitate the introduction of novel pharmacological interventions into clinical practice, limit the adverse effects of drug cargo, and improve the efficiency of targeted delivery [56]. MRNPs have the potential to increase the resistance to detergents, Enz inhibitors, metal ions, pH, and temperature, prolong the Enz activity, present long-term stability, and facilitate delivery through the cell membrane [82, 83].

The effectiveness of antioxidant Enzs for combating oxidative stress depends on the ability to obtain a therapeutically sufficient level of an active Enz at reactive nitrogen/oxygen species (RNS/ROS)-induced injury. Therefore, the accomplishment of antioxidant Enz therapy needs an approach enabling efficient safeguarding of the Enz in its active form and directed transfer to the intended location [84]. Chorny et al. established MRNPs as a nanocarrier for the magnetically directed transfer of therapeutic protein to address these issues. The SOD and CAT-loaded NPs displayed a prolonged release of their cargos in plasma. CAT-loaded NPs were preserved from proteolysis. They maintained 25% of their primary enzymatic activity after one day of exposure to a commercially available mixture of extracellular Enzs. These MRNPs were also taken up rapidly via endothelial cells, enhancing oxidative stress resistance [55]. Although the development of MRNPs for the directed transfer of gene vectors and small pharmaceuticals has been improved [85], the therapeutic potential of NPs delivery for site-specific targeting of biologically active Enzs/proteins has mainly remained unknown to significant difficulties in the insufficient Imb or unsuitable formulation.

#### Metallic (gold and silver) NPs

Gold (Au) NPs are considered promising Enz/protein delivery candidates due to their cellular diagnosis feature and non-toxicity nature [86]. The tunable functionality and large surface area of AuNPs make them an outstanding scaffold for Enz/protein surface recognition [47, 87, 88]. Also, efficiently delivering lysosome storage complications caused by insufficient lysosome-impermeable and negatively charged Enz. The ANPs transfect various cell lines with functional Enzs, allowing them to escape the endosomal trapping and maintain their enzymatic activity. Moreover, AuNPs did not influence Enz activity; however, the amount of protein and size of the NPs may affect the enzymatic activity of the conjugate [89]. However, inorganic NPs, AuNPs, can lead to health problems in the long term due to the presence of heavy metals in their composition.

Silver (Ag) NPs indicate unique features, including tunable physicochemical profile, surface plasmon resonance, and surface functionalization [90, 91]. The bio-conjugation of Enzs to AgNPs with various biomolecules (peptides, nucleic acid, and Enzs) mitigates the NP toxicity and regulates their cellular uptake for enhanced in vivo activity [92]. Recently, AgNPs have been used to stabilize Enzs for biosensor production, biodiesel, and, more recently, drug production. Likewise, Ernest et al. [50] observed that AgNPs-LYZ nanoconjugates possess synergistic antibacterial properties without affecting the catalytic site of LYZ [50]. In another study, trypsin was stabilized on AgNPs to increase protein digestion, and immobilized trypsin revealed more stability, activity, and hydrolyzing capacity than the free counterpart [51, 93]. Furthermore, in another study, Imb of βKT on AgNPs supports enhanced dehairing activity, antibacterial potency, and organoleptic analysis in the pharmaceutical and cosmetic industries [52].

### Polymeric nanoparticles

Natural polymers, including chitosan (CS), collagen, cellulose, chitin, creatine, and alginate and synthetic polymers, including poly lactic-co-glycolic acid (PLGA), poly lactic acid (PLA), polyethylene glycol (PEG), polycaprolactone (PCL), polyvinyl pyrrolidone (PVP) and poly acrylic acid (PAA) were utilized for the preparation of polymeric NPs [94]. These polymers have several advantages, including better control over payload release, more targeted transport, enhanced cellular uptake, decreased side effects, and improved payload bioavailability by reducing the degradation rate of Enz delivery [61, 94]. Likewise, it was found that immobilizing the Enz in polymeric NPs doesn’t influence the Enz conformation, function, and activity [95]. Therefore, the biocompatibility, biodegradability, and cost-effectiveness of polymeric NPs make them an excellent source of Enz and can be re-used numerous times [96]. Besides, many organs display a particular pH, and by tuning the contact duration and degree of the electrostatic interactions between the charged polymeric NPs and target organs, long-term therapeutic efficacies and determination of transfer routes can be achieved in Enz delivery [97]. To date, the stabilization in the carriers and the design of suitable polymeric NPs to overcome the mucosal barrier have been explored. A study demonstrated that NPs made with CS and insulin provided a long-term and high systemic immune response and allowed for the active transport of encapsulated Enzs across the nasal and intestinal mucosae after intranasal administration [60]. However, polymeric NPs also have indicated disadvantages in EI and Enz delivery with the increase in immunogenicity during decomposition, which is caused by possible impurities in natural polymers. In addition, the possibility of creating acidic compounds due to degradation may cause problems [98].

### Lipid-based NPs

Liposomes have been widely used as a carrier to deliver both hydrophilic and hydrophobic drugs, antibacterial and antifungal agents, vaccines, Enzs, and genetic cargo [99]. This popularity refers to high drug/lipid ratio loading efficiency, ease of fabrication in a size-controlled manner, long-circulating by polyethylene glycol (PEG) coating, excellent stability, controllable release kinetics, and biocompatibility [100]. Liposomal formulations have shown an ability to enhance the PK and PD of active pharmaceutical ingredients (API), while hindering their associated off-target toxicity [101]. To explore this role, Chen et al. [67] investigated the therapeutic effects of papain elastic liposomes (PEL) on hypertrophic scar through skin delivery. They revealed that PEL is excellent topical preparation for hypertrophic scar treatment in vitro and in vivo. Moreover, the scar elevation index, microvascular density, and collagen fiber were significantly decreased. Finally, the expressions of TGF-β_1_, P-Smad-3, P-NF-κB p65, and P-IKBa in hypertrophic scar were significantly down-regulated in contrast with those in the model group.

### Biological NPs

Despite being less studied, protein NPs, an important class of biological NPs, offer several benefits over their liposomal, inorganic, and polymeric counterparts, such as amino-acid degradation products, surrounding protein environment, ease of production, and their high loading [102, 103]. Despite the optimization of NPs (crystallization and encapsulation), their large size prevents them from being effectively delivered to cells. The activity of linked Enz crystals like glucose oxidase-loaded protein particles was higher than EI on the surface of AuNPs, which Enz activity was maintained after intracellular delivery.

Red blood cells (RBCs), another class of biological NPs, are proposed as a novel promising DDS. RBCs represent a low or virtually no immune response due to their biocompatibility, which protects the immobilized substances from rapid clearance and prevents toxic side effects. In addition, RBC can be readily obtained, and a large quantity of the drugs can be entrapped into a relatively small cell volume using specific well-defined methods [104, 105]. Exogenous elements can encapsulate into RBCs via endocytosis, passive diffusion, electroporation, and hypotonic dialysis/isotonic resealing techniques. Specific Enzs represented more potential as cargo for them than non-enzymatic antidotes, including glutathione [106]. To date, more than 10 various Enzs have been encapsulated into RBCs [107]. These Enzs cover alcohol oxidase and alcohol dehydrogenase for the elimination of methanol and other toxic alcohols [74]. Furthermore, phosphodiesterase to antagonize organo-phosphorus compounds (toxin paraoxon) [75], thiosulfate-cyanide sulfurtransferase (AKA rhodanese), to convert cyanides into less toxic thiocyanates [76], and uricase to eliminate uric acid [77],[108] were utilized.

While many inorganic NPs have been developed as carriers, few have entered clinical studies, compared to polymers or liposomes. Comparatively to organic materials, inorganic NPs have several advantages, including non-toxic, hydrophilic, biocompatible, and highly stable properties. On the other hand, inorganic materials usually have a smaller particle size, better stability, controlled tunability, greater permeability, and a higher antigen loading than organic materials [109]. It is becoming increasingly important, particularly for treating gastroenterological (GI) illnesses locally. Theranostic applications of inorganic NPs, which help diagnose and treat the same patient, are the most frequently explored [110]. It is possible to categorize polymeric nanoparticles into nanospheres and nanocapsules, which are excellent DDS. Similarly, phospholipid nanostructures, liposomes, and micelles are extremely useful in targeted drug delivery. The composition and properties of lipid and polymeric NPs make them the most suitable candidates for pediatric applications [111]. Compared to other NPs, the materials used to make them impart to NPs biocompatibility, biodegradability, non-immunogenicity and non-toxicity, and a high drug entrapment efficiency. Polymeric and lipid-based NPs are the most clinically approved NPs in EI. The NP manipulation as hybrid NPs (polymer-lipid and etc.) may be a promising approach to delivering EI precisely and effectively [112].

## Conclusion and future perspectives

Enz therapy offers a novel and promising orientation for the next generation of proteins-mediated treatments. Albeit most targets of US FDA-approved enzymatic drugs are extracellular, there are different diseases for which treatment needs intracellular delivery. This requires intracellular delivery by efficient tools such as NPs. More therapeutic uses of enzymatic drugs are expected to emerge, including the reversal of phosphorylation of Tau protein for Alzheimer disease. Enzymatic drugs can conduct these functions effectively, but they need encapsulation, Imb, or modification to improve their distribution and stability. Overall, our review provides a comprehensive framework to develop the nanosystems that improve the delivery process of enzymatic drugs to target tissues or cells. Therefore the utilization of the ideal nanosystem for drug delivery is determined predominately according to the biochemical and biophysical features of the intended drugs being chosen for the therapy [113]. However, toxicity displayed by NPs cannot be passed over when concerning the usage of therapeutic Enzs [114]. Among NPs, cellulose, chitosan, graphene, gold, magnetic, silica, and titanium NPs indicate the predominant potential for the delivery of clinical Enz [4, 115–117].

Recently, NPs have been combined with natural metabolites to reduce cytotoxicity problems. The green chemistry-based design of drug-loaded NPs is greatly encouraged because it minimizes the hazardous compounds in the biosynthetic processes [41]. Therefore, the application of green NPs for Enz delivery can diminish the side-effect of the drugs. Besides, the adjustment of surface changes, hydrophobicity, shape, and nanostructure size can further improve the bioactivity of these nanosystems.

The use of nanotherapeutics has recently been found to be the most attractive approach to both the treatment and diagnosis of clinical complications [118]. In order to deliver an Enz precisely, such as to cancer cells, without interrupting the normal physiology of the cells, a variety of nanoparticles can be used to deliver an Enz at precise levels. The use of NPs-mediated Enz delivery is unquestionably the trend that will remain the future area of research and development for years to come [118]. On the other hand, manipulation of new material for EI decreases the conventional heterogenicity of utilized Enz approaches. Furthermore, concomitant solid supported material for immobilized Enzs, along with the development of NPs, increases the Enz features like activity, targeting, efficacy, and stability. Moreover, this modification decrease toxicity, side effect, and off-target for Enz performance. Subsequently, understanding and select of the material for fabrication and design new supporting approach depends on the Enz type in the Imb strategies, preparation of hybrid NPs to promotion of compatibility and efficiency of carrier facilities Enz therapeutics to promise emerging way into clinical development.

## Data Availability

Not applicable.
